# CD11c^+^ CD8^+^ T Cells Reduce Renal Fibrosis Following Ureteric Obstruction by Inducing Fibroblast Apoptosis

**DOI:** 10.3390/ijms18010001

**Published:** 2016-12-22

**Authors:** Haidong Wang, Juan Wang, Yun Bai, Jinwei Li, Lixin Li, Yanjun Dong

**Affiliations:** 1 College of Veterinary Medicine, China Agricultural University, Haidian, Beijing 100193, China; whd1232123@163.com; 2 College of Animal Science and Veterinary Medicine, Shanxi Agricultural University, Jinzhong 030801, China; by15110692544@163.com (Y.B.); 18335442405@163.com (J.L.); m15534992431@163.com (L.L.); 3 Department of Geriatrics, Tongji Hospital of Shanghai affiliated to Tongji University, Shanghai 20065, China; wangjunshanghai@163.com; 4 The Beijing Institute of Heart Lung and Blood Vessel Diseases, Chaoyang, Beijing 100029, China

**Keywords:** T cells, fibroblasts, fibrosis, inflammation, kidney

## Abstract

Tubulointerstitial fibrosis is a common consequence of various kidney diseases that lead to end-stage renal failure, and lymphocyte infiltration plays an important role in renal fibrosis. We previously found that depletion of cluster of differentiation 8^+^ (CD8^+^) T cells increases renal fibrosis following ureteric obstruction, and interferon-γ (IFN-γ)-expressing CD8^+^ T cells contribute to this process. CD8^+^ T cells are cytotoxic T cells; however, whether their cytotoxic effect reduces fibrosis remains unknown. This study showed that CD8^+^ T cells isolated from obstructed kidney showed mRNA expression of the cytotoxicity-related genes *perforin 1*, *granzyme A*, *granzyme B*, and *FAS ligand*; additionally, CD8 knockout significantly reduced the expression levels of these genes in obstructed kidney. Infiltrated CD8^+^ T cells were distributed around fibroblasts, and they are associated with fibroblast apoptosis in obstructed kidney. Moreover, CD11c^+^ CD8^+^ T cells expressed higher levels of the cytotoxicity-related genes than CD11c^−^ CD8^+^ T cells, and infiltrated CD11c^+^ CD8^+^ T cells in obstructed kidney could induce fibroblast death in vitro. Results indicated that induction of fibroblast apoptosis partly contributed to the effect of CD8^+^ T cells on reduction of renal fibrosis. Given that inflammatory cells are involved in fibrosis, our results suggest that kidney fibrosis is a multifactorial process involving different arms of the immune system.

## 1. Introduction

Tubulointerstitial fibrosis is the common end point of most progressive chronic kidney diseases (CKD) [1] and is accompanied by widespread lymphocyte infiltration into the kidney. Excessive amount of T cells have been found in the kidneys of CKD patients [2] and in mouse models of renal fibrosis, such as in unilateral ureteric obstruction (UUO) models [3,4,5,6,7]. UUO-induced T cell infiltration and fibrosis in kidney can be reduced by knockout of C–C chemokine receptor type 1 (CCR1) or by blockage of CCR1 function [4,7]. Studies have shown that cluster of differentiation 4^+^ (CD4^+^) and CD8^+^ T cells are implicated in UUO-induced fibrosis. Reconstitution of lymphopenic immune-deficient *recombination activating gene* (RAG) knockout mice with purified CD4^+^ T cells prior to ureteric obstruction significantly increased the interstitial expansion and collagen deposition [6] We previously found that depletion of CD8^+^ T cells increases renal fibrosis following ureteric obstruction, and interferon-γ (IFN-γ)-expressing CD8^+^ T cells contribute to this process [8]. CD8^+^ T cells are cytotoxic T cells; however, whether their cytotoxic effect contributes to reduction of fibrosis remains unknown.

Development of cytotoxic T cells requires CD8 protein compared with that of helper T cells. The absence of surface expression of CD8 resulted in the lack of mature cytotoxic T cells, whereas this phenomenon did not affect the number of helper T cells. CD8^+^ T cells, which usually exist as cytotoxic T lymphocytes, serve as killer cells by lysing their target cells; by contrast, CD4^+^ T cells, which are helper cells, produce lymphokines and promote activation and/or proliferation of B cells, cytotoxic T lymphocytes, and macrophages [9]. Some key proteins in CD8^+^ T cells can kill target cells through direct contact with these target cells. These proteins are cytoplasmic granule toxins, popularly known as perforin and granzymes; these proteins are secreted via exocytosis and together they induce the apoptosis of their target cells [10,11]. Another protein is FasL, which is a component of the FasL-Fas system, which induces caspase-dependent apoptosis [12].

Our previous study has indicated that depletion of CD8^+^ T cells exacerbates CD4^+^ T cell-induced monocyte-to-fibroblast transition in renal fibrosis. The present results indicated that induction of fibroblast apoptosis partly contributes to the ability of CD8^+^ T cells to reduce renal fibrosis.

## 2. Results

### 2.1. CD8 Deficiency Promotes Renal Fibrosis in Unilateral Ureteric Obstruction (UUO) Mouse Model

Using a ureteric obstruction model, Thomas et al. have discovered a critical role of CD4^+^ T cells in kidney fibrosis in RAG^−/−^ mice [6]. Additionally, they investigated the role of CD8^+^ T cells in kidney, but they could not explain the function of CD8^+^ T cells in renal fibrosis using their model.

**Figure 1:**
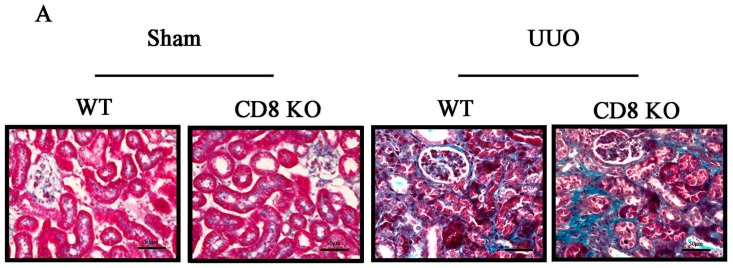

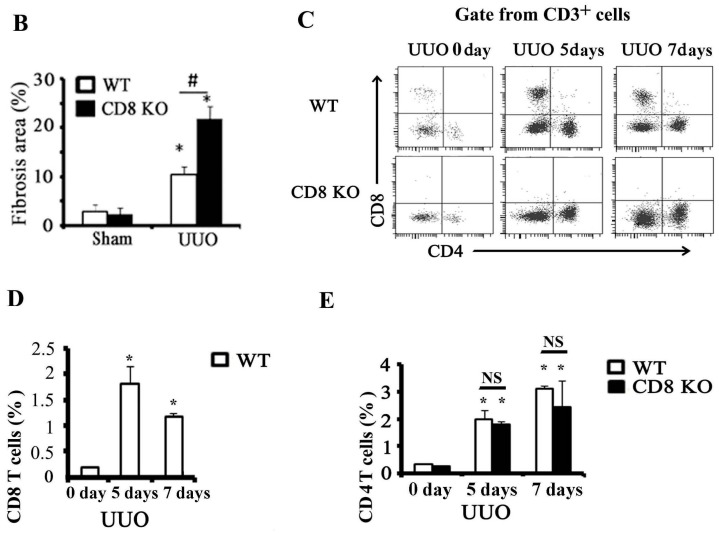
CD8 deficiency promotes renal fibrosis in unilateral ureteric obstruction (UUO) mice. (**A**) Masson’s Trichrome staining was performed to examine fibrosis at day 7; (**B**) Quantitative analysis of fibrosis area in UUO kidneys. CD8 knockout (KO) increases fibrosis in UUO kidney (* *p* < 0.05 vs. sham, # *p* < 0.05 vs. wild-type (WT) UUO; *n* = 5/group). Scale bars, 50 µm; (**C**) In fluorescence-activated cell sorting analysis, kidney CD8^+^ T cells were stained with anti-CD45-PerCP-Cy5.5, anti-CD3e-PE-Cy594, anti-CD8-APC-Cy7, and anti-CD4-PE to confirm the absence of CD8^+^ cells in CD8 KO mice and quantitate (**D**) CD8^+^ T cells or (**E**) CD4^+^ T cells. (* *p* < 0.05 vs. WT UUO 0 day, NS: no significant difference; *n* = 6/group).

Our previous study has indicated that CD8^+^ T cell depletion exacerbates CD4^+^ T cell-induced monocyte-to-fibroblast transition in renal fibrosis [8]. On the basis of the study above, we performed UUO surgery in C57BL/6 and CD8 knockout (KO) mice and then collected their kidneys at days 0, 5, and 7; deposition of renal tubular interstitial collagen was quantified in a section stained with Masson’s Trichrome stain (Figure 1A, which shows the sample obtained at day 7). The fibrosis area at day 7 is markedly higher in UUO mice than in sham-operated mice. Moreover, the number of CD8^+^ cells increased in kidney after UUO, and the number of CD8^+^ cells peaked at day 5 (Figure 1C,D). Depletion of CD8^+^ T cells did not affect the number of CD4^+^ T cells (Figure 1C,E), but it increases renal fibrosis at UUO day 7 (Figure 1A,B). These results showed that CD8 deficiency increases renal fibrosis.

### 2.2. Activation of CD8^+^ T Cell Requires an Inflammatory Microenvironment

**Figure 2:**
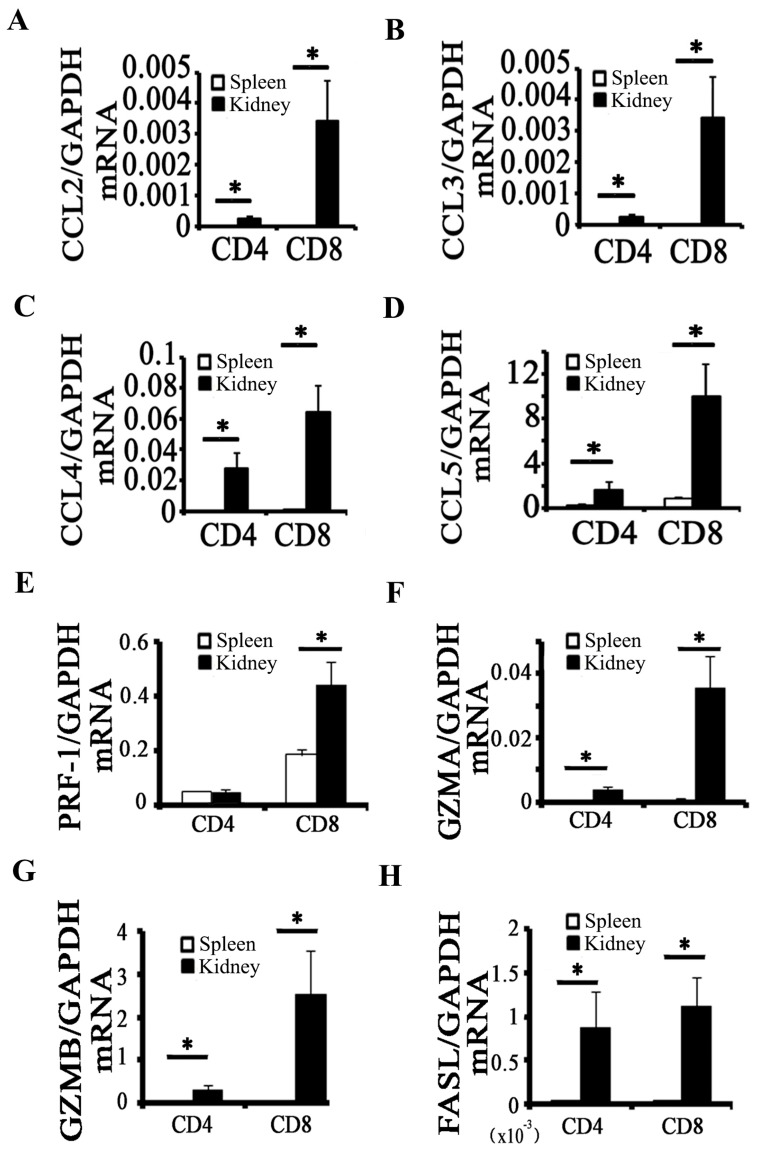
CD8^+^ T cell activation requires an inflammatory microenvironment. CD4^+^ T cells (CD45^+^ CD3^+^ CD4^+^ CD8^−^) and CD8^+^ T cells (CD45^+^ CD3^+^ CD4^−^ CD8^+^) were isolated from obstructed kidney or spleen. (**A**–**D**) mRNA levels of chemokines (CCL2, CCL3, CCL4, and CCL5) as determined in those CD4^+^ T cells or CD8^+^ T cells by quantitative RT-PCR (qRT-PCR; * *p* < 0.05 kidney vs. spleen; *n* = 5); (**E**–**H**) mRNA levels of cytotoxicity-related genes (*PRE-1, GZMA, GZMB, and FASL*) as determined in those CD4^+^ T cells or CD8^+^ T cells by qRT-PCR (* *p* < 0.05 kidney vs. spleen; *n* = 5).

Chemokines are important factors in the recruitment of inflammatory cells. To examine whether CD8^+^ T cells contribute in chemokine expression in obstructed kidney, we measured the mRNA expression levels of primary CCLs (CCL2, CCL3, CCL4, and CCL5) in CD8^+^ T cells isolated from obstructed kidneys or spleens. The obstructed kidneys were harvested from UUO 5 days mice to separate CD4^+^ T cells and CD8^+^ T cells through cell sorting; T cells separated from spleens served as control. CD8^+^ T cells and CD4^+^ T cells in obstructed kidney expressed CCL2, CCL3, CCL4, and CCL5 compared with those in spleen; moreover, mRNA expression levels of these chemokines were higher in CD8^+^ T cells than in CD4^+^ T cells (Figure 2A–D). Despite this disparity, the mRNA expression levels of CCL2, CCL3, CCL4, and CCL5 in obstructed kidney of CD8 KO and wild-type (WT) mice did not significantly differ [8]. To test whether CD8^+^ T cells produce cytotoxicity factors in obstructed kidney, we isolated CD8^+^ T cells from obstructed kidney to examine the mRNA expression levels of cytotoxicity-related genes, namely, *perforin 1* (PRF-1), *granzyme A* (GZMA), *granzyme B* (GZMB), and *FAS ligand* (FASL); CD8^+^ T cells isolated from spleen served as control. The result showed that expression levels of PRF-1, GZMA, GZMB, and FASL were higher in CD8^+^ T cells of obstructed kidney than those in spleen (Figure 2E–H).

### 2.3. CD8 Deficiency Reduces Cytotoxicity and Cell Apoptosis in Obstructed Kidney

**Figure 3:**
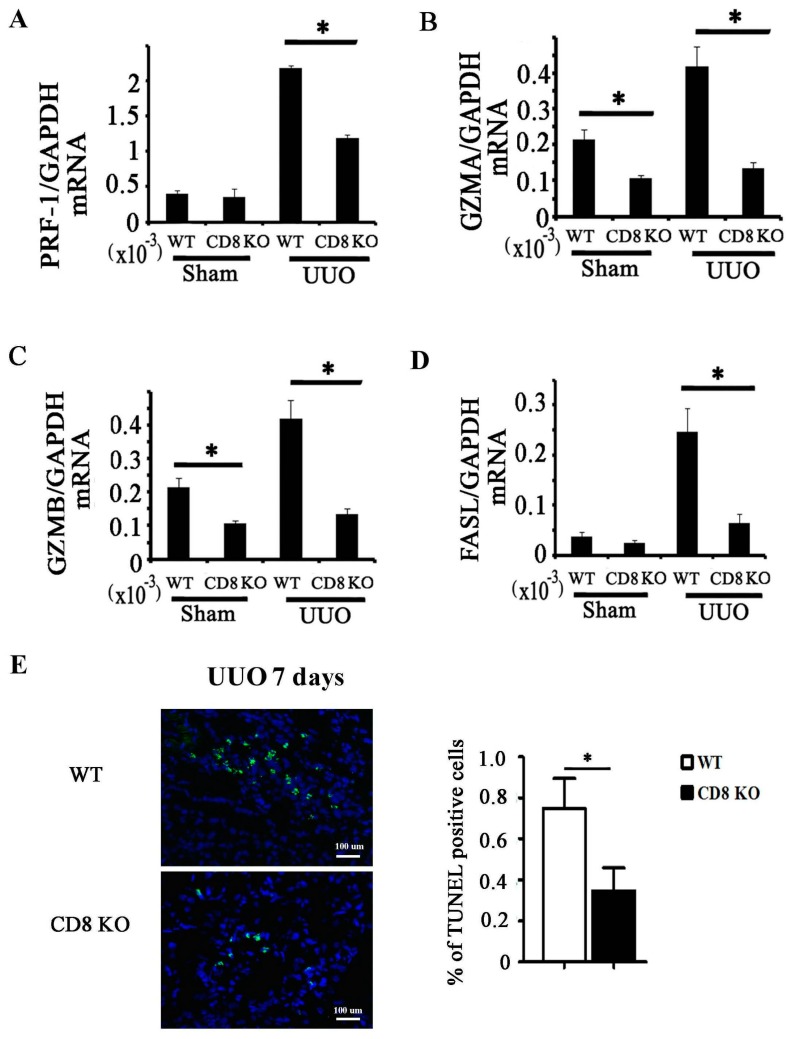
CD8 KO reduces cytotoxicity and cell apoptosis in obstructed kidney. (**A**–**D**) mRNA expression levels of PRF-1, GZMA, GZMB, and FASL in whole kidney tissues were analyzed by quantitative RT-PCR (* *p* < 0.05, CD8 KO vs. WT; *n* = 5); (**E**) TUNEL staining is performed in kidney; TUNEL (green), nucleus (blue). The percentage of TUNEL-positive cells in total cells is calculated (*n* = 5, * *p* < 0.05).

Under CD8 deficiency, the mRNA expression levels of cytotoxicity-related genes decreased in obstructed kidney (Figure 3A–D). Cytotoxicity factors are related to cell apoptosis, so apoptosis in WT or CD8 KO kidney with UUO was examined through TUNEL staining. We found that CD8 deficiency significantly reduced cell apoptosis in obstructed kidney (Figure 3E).

### 2.4. CD11c^+^ CD8^+^ T Cell Induces Fibroblast Death

**Figure 4:**
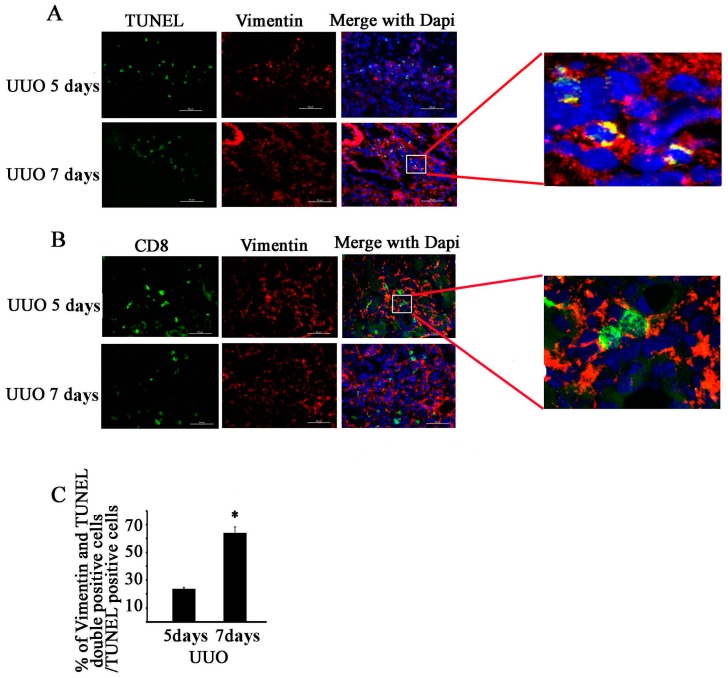
CD8^+^ T cells are associated with apoptosis of fibroblast in obstructed kidney. (**A**) Double immunostaining in kidney was performed using anti-vimentin (red) and TUNEL (green) at UUO 5 or 7 days (bar = 50 µm); (**B**) Double immunostaining in kidney was performed using anti-vimentin (red) and anti-CD8 (green) at UUO 5 or 7 days (Bar = 50 µm); (**C**) Percentage of vimentin- and TUNEL-double positive cells among TUNEL-positive cells was calculated (* *p* < 0.05; *n* = 5).

Fibroblasts are the main sources of renal fibrosis in UUO model [13]. We used TUNEL and vimentin antibody to perform double immunofluorescence staining of a section of obstructed kidney at UUO (5 and 7 days) (Figure 4A); vimentin is a fibroblast cell marker. The results showed the 24% ± 2% of apoptotic cells at UUO 5 days and 65% ± 4% at UUO 7 days were fibroblasts (*n* = 5, *p* < 0.05, Figure 4C). CD8 and vimentin were located in the same region; CD8-positive cells were distributed around vimentin-positive cells either at UUO 5 or 7 days (Figure 4B).

**Figure 5:**
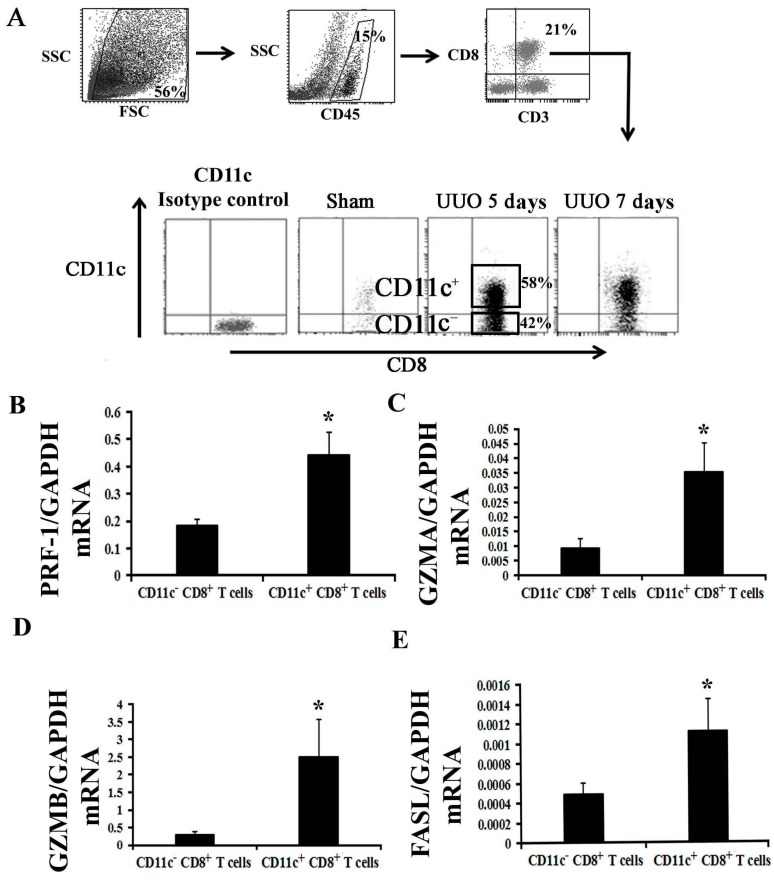
CD11c^+^ CD8^+^ T cells mainly constitute the population of CD8 T cells expressing the mRNA of cytotoxin related factors in obstructed kidney. (**A**) CD11c^−^ CD8^+^ T cells and CD11c^+^ CD8^+^ T cells in kidney at UUO 5 days were separated through cell sorting after incubation of the total number of cells in antibody cocktail (anti-CD45, CD3e, CD8a, and CD11c). CD11c isotype control group: incubated in antibody cocktail (anti-CD45, CD3e, and CD8a and an isotype control of CD11c antibody); (**B**–**E**) mRNA levels of cytotoxin related factors (PRE-1, GZMA, GZMB, and FASL) as determined in those CD11c^−^ CD8^+^ T cells or CD11c^+^ CD8^+^ T cells by quantitative RT-PCR (* *p* < 0.05 vs. CD11c^−^ CD8^+^ T cells; *n* = 5).

To demonstrate whether CD8^+^ T cells can kill fibroblasts, we isolated CD8^+^ T cells from obstructed kidney, and we subsequently co-cultured them with fibroblasts to determine the fibroblast mortality. Zhubo et al. have identified an infection-induced new subset of CD11c^high^ CD8^+^ regulatory T cells, which possibly protect a host from pathological immune injury [14]. Whether cells similar to CD11c^high^ CD8^+^ T cells exist in obstructed kidney is unknown; we isolated these cells to examine their function. Unfortunately, we could not find cells that are exactly similar to CD11c^high^ CD8^+^ T cells in CD11c^+^ CD8^+^ T cells in obstructed kidney at UUO 5 or 7 days. However, two subsets of CD8 T cells were classified from CD11c^+^ CD8^+^ T cells according to their CD11c expression; these subsets are the CD11c^low^ CD8^+^ and CD11c^high^ CD8^+^ T cells in Zhoubo′s model; thus, we simply separated CD11c^+^ CD8^+^ and CD11c^−^ CD8^+^ T cells (Figure 5A).

**Figure 6:**
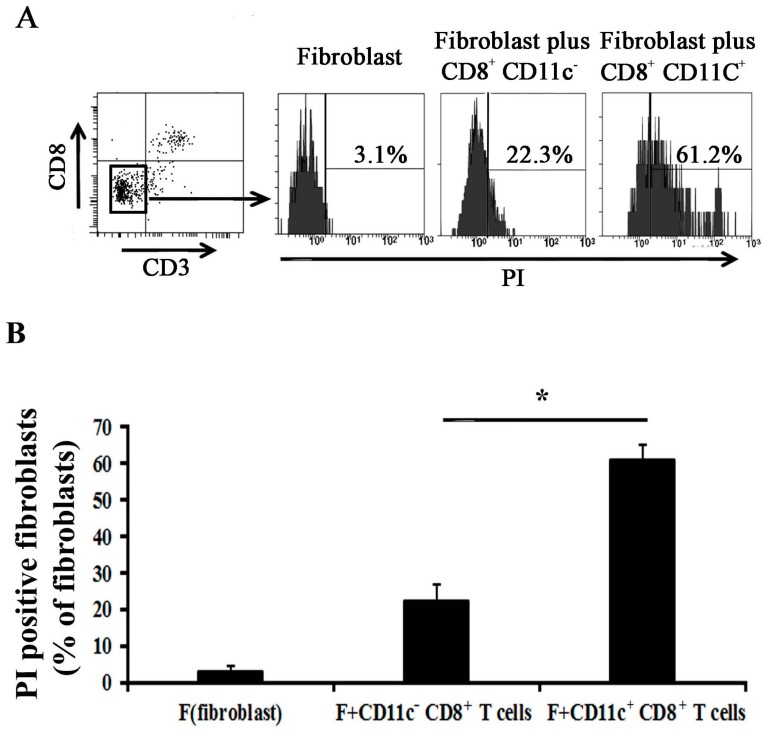
CD11c^+^ CD8^+^ T cells isolated from obstructed kidney can induce fibroblast death in vitro. (**A**) Fibroblasts were co-cultured with CD11c^−^ CD8^+^ T cells or CD11c^+^ CD8^+^ T cells for 24 h; the cells were subsequently harvested to analyze the mortality of CD8^−^ CD3^−^ cells (fibroblast) through PI (Propidium Iodide) staining combined with flow cytometry; the CD8^+^ T cell-free set-up served as control; (**B**) Quantitative analysis of the percentage of PI staining-positive fibroblasts. (* *p* < 0.05 vs. fibroblasts plus CD11c^−^ CD8^+^ T cells; *n* = 5/group).

To examine which subset population of CD8^+^ T cells mainly produces the cytotoxin related factors, we examined the mRNA expression levels of PRE-1, GZMA, GZMB, and FASL. The results indicated that the CD11c^+^ CD8^+^ T cells mainly produce the cytotoxin related factors (Figure 5B–E). We then cultured the CD11c^+^ CD8^+^ or CD11c^−^ CD8^+^ T cells with fibroblast, and the results showed that the mortality of fibroblasts in fibroblast alone group is 3.1% ± 1.2%; 22.3% ± 4.6% in fibroblast plus CD11c^−^ CD8^+^ group, and 61.2% ± 3.9% in fibroblast plus CD11c^+^ CD8^+^ group. Either CD11c^+^ CD8^+^ or CD11c^−^ CD8^+^ T cells could induce death of fibroblasts, and the effect of CD11c^+^ CD8^+^ T cells was higher than that of CD11c^−^ CD8^+^ T cells (*p* < 0.05, *n* = 5) (Figure 6).

## 3. Discussion

Lymphocyte infiltration and renal fibrosis are widespread in most progressive kidney diseases. Understanding the relationship between lymphocyte infiltration and renal fibrosis may lead to prevention of the progression of kidney diseases and renal failure.

CD8^+^ T cell infiltration increased following UUO, and renal fibrosis was obvious at day 7 (Figure 1). We used CD8 KO mice to determine whether CD8 T cell infiltration would affect the severity of renal fibrosis after UUO. The result showed that CD8 T cell depletion reduces renal fibrosis after UUO, but the mechanism remains unknown. CD4 T cells [6], macrophages [15], neutrophils [16], and other inflammatory cells contribute to organ fibrosis; moreover, CD8 T cells contribute to macrophage recruitment by CCL2 in a muscle fibrosis model [17]. To examine whether CD8 T cells contributed to the recruitment of inflammatory cells in kidney by secreting chemokines, we compared the expression levels of CCL2, CCL3, CCL4, and CCL5 in kidney and spleen. The results showed that the CD8^+^ T cells in kidney expressed higher mRNA levels of chemokine genes than the CD8^+^ T cells in spleen after UUO (Figure 2). However, our previous study showed that CD8 KO in mice does not affect total chemokine levels in kidney compared with those in WT mice [8]. Our results suggest that CD8^+^ T cells are not the only type of cells that secrete chemokines; other secretory cells will secrete more chemokines to compensate for the absence of CD8 T cells after UUO. More importantly, both CD8^+^ T cells and CD4^+^ T cells originate from CD8^+^ CD4^+^ T cells; thus, CD8 KO affects the development and infiltration of CD4^+^ T cells. A study using CD8 KO mice showed that CD8 KO does not affect the development of helper T cells [9]; additionally, our result showed that CD8 KO does not affect CD4 T cell infiltration (Figure 1E). Thus, we speculated that CD8 KO could not affect the recruitment of inflammatory cells in kidney after UUO.

Tapmeier’s results [6] on variation in renal fibrosis after reconstitution of RAG^−/−^ mice with CD8^+^ T cells do not conclusively show that CD8^+^ T cells exert effect on renal fibrosis. However, we previously observed a significantly higher level of fibrosis and upregulated α-SMA and fibronectin at UUO 7 days in CD8 KO mice compared with those in WT [8]. Tapmeier speculated that their result did not exclude the effect of CD8^+^ T cells, because reconstituted CD8^+^ T cells may lack cooperation of CD4^+^ T cells to elicit their effect in vivo. Our result showed that the functions of CD8 cells not only requires the participation of CD4^+^ T cells but also requires an inflammatory microenvironment, as indicated by the mRNA levels of chemokines and cytotoxin related factors (Figure 2) in CD8^+^ T cells isolated from kidney after UUO 7 days compared with the mRNA levels in CD8^+^ T cells isolated from spleen.

CD8^+^ T cells are usually cytotoxic T lymphocytes responding to antigenic challenge by lysing their target cells. CD8^+^ T cells secreted cytotoxin related factors in kidney after UUO (Figure 2E–H), and CD8 KO significantly reduced the total levels of cytotoxin related factor and cell apoptosis in kidney relative to those in WT (Figure 3). We subsequently determined which cell type had undergone apoptosis and thus had contributed to the reduction of fibrosis in the kidney. Fibroblasts are the main origin of fibrosis in UUO model; thus, we examined the relationship between CD8^+^ T cell and fibroblast apoptosis in obstructed kidney. Our result showed that CD8^+^ T cells gather around the fibroblasts (Figure 4B), and fibroblast apoptosis increased gradually (Figure 4A,C) following renal fibrosis. We speculated that CD8^+^ T cell infiltration killed some of the fibroblast, reducing fibrosis.

Studies have shown that T cells induce fibroblast apoptosis under a certain pathologic condition. For example, a CD8^+^ T cell recognizes fibroblast activation protein to kill tumor-associated fibroblasts [18]; peripheral T cells of patients with early systemic sclerosis kill autologous fibroblasts in co-culture [19]; and γδ T cell induces fibroblast apoptosis in injured liver [20]. Zhubo et al. have recently identified an infection-induced new subset of CD11C^high^ CD8^+^ regulatory T cells, which possibly contribute in protecting the host from pathological immune injury [14]. In their model, two subsets of CD8 T cells were classified according to CD11c expression; these subsets are CD11c^low^ CD8^+^ and CD11c^high^ CD8^+^ T cells. CD11c^low^ CD8^+^ T cells, which exist during the entire period of infection, act as conventional activated T cells that kill target cells in a perforin-dependent manner. We attempted to isolate these cells from an obstructed kidney but we could only isolate CD11c^+^ CD8^+^ T cells and CD11c^−^ CD8^+^ T cells (Figure 5A) possibly because CD11c^high^ CD8^+^ T cells only appear at a late stage of infection but not in ureteral obstruction, which is a pathological process not involving an infection. Our data showed that CD8^+^ T cells isolated from obstructed kidney could kill fibroblast in vitro. CD11c^+^ CD8^+^ T cells play a main role in killing fibroblast, although CD11c^−^ CD8^+^ T cells can also kill fibroblasts (Figure 6).

## 4. Materials and Methods

### 4.1. Animals and Surgery

Male CD8 KO mice (B6.129S2-Cd8a^tm1Mak/J^) and WT mice (C57BL/6J) were obtained from Jackson Laboratory (Bar Harbor, ME, USA). The mice were 10–12-week old and reared under a 12 h light/12 h dark cycle. The mice were provided with a standard diet. These mice were subjected to UUO as described previously [8]. In brief, UUO operation was performed after induction of general anesthesia through intraperitoneal injection; the anesthesia was a combination of 12 mg/kg xylazine and 60 mg/kg ketamine; a midline incision was made and the left ureter was exposed and tied off with 4-0 silk suture at two points. Sham operation was performed in the same manner but without ureter ligation. All mice were sacrificed at 0, 5, or 7 days after UUO operation, and the kidneys were harvested for analyses. All protocols for animal care and experimentation complied with the Animal Management Rule of the Ministry of Health, China (Documentation No. 55, 2001) and the Guide for the Care and Use of Laboratory Animals published by the US National Institutes of Health (NIH Publication No. 85-23, revised 1996).

### 4.2. Histology and Imaging

After anesthesia, the mice were perfused with PBS through the left ventricle. Their kidneys were removed and processed for cryosectioning or paraffin sectioning. The sections were stained with Masson’s Trichrome [21]. The severity of interstitial renal fibrosis was evaluated by calculating the positive area of blue staining of total tissue measured using the NIS-ELEMENTS quantitative automatic program (Nikon, Tokyo, Japan). Immunostaining was performed using antibodies against Vimentin (Abcam, Cambridge, MA, USA) and CD8a (Becton Dickenson, Franklin Lakes, NJ, USA). In brief, the sections were permeabilized with 0.3% Triton X-100 in PBS and blocked with protein block (DakoCytomation, Glostrup, Denmark) for 1 h at room temperature. The sections were incubated with a primary antibody mixed in “Antibody Dilute” (DakoCytomation) followed by detection of the primary antibodies using any second antibodies conjugated to Alexa 568 or 488 (Invitrogen, Eugene, OR, USA). TUNEL staining was performed using a Fluorometric TUNEL System (Promega, Madison, WI, USA). Tissues were visualized using a Nikon 80i microscope, and images were obtained using a DS-cooled camera and the NIS-Elements Br 3.0 software (Nikon, Melville, NY, USA). To quantify the number of TUNEL positive cells or vimentin^+^ TUNEL^+^ cells in obstructed kidney, we imaged five fields in cortex renis of each mouse, and we analyzed five mice in each group.

### 4.3. RT-PCR Analysis

RT-PCR was performed as previously described, and relative gene expression was calculated from cycle threshold (*C*_t_) values using GAPDH as internal control (relative expression = 2^(GAPDH *C*t − sample *C*t)^) [22]. All samples were run in duplicate. Primers and their sequences are listed in Table 1.

### 4.4. Tissue Preparation, Fluorescence-Activated Cell Sorting, and Flow Cytometry

Kidneys and spleens were dissected and ground separately. The kidney fragments were digested with 2 mL of collagenase type IA (2.5 U·mL^−1^, Sigma Chemical Company, St. Louis, MO, USA) in PBS containing 10 mM CaCl_2_ at 37 °C for 30 min. After being washed, the kidney and spleen slurries were passed separately through a 40-μm strainer (BD Biosciences, Franklin Lakes, NJ, USA) and then washed with PBS. Cells were collected by centrifugation at 1500 rpm for 5 min and incubated in PBS containing 2 mM EDTA and 2% FBS plus primary antibodies for 30 min at 4 °C. The cells were resuspended at 1 × 10^7^ cells/mL before sorting or analysis. The cells were separated and analyzed on BD FACSAria II or BD FACSCanto II (BD Biosciences) by Beijing Institute of Heart, Lung and Blood, Vessel Diseases Cytometry and Cell Sorting Core Facility, and data were collected using FACSDiva 7.0 software (BD Biosciences). The antibodies used were anti-CD4-PE (Clone RM4-5), anti-CD3e-PE-cf594 (Clone 145-2C11), anti-CD8a-APC-cy7 (Clone 53-6.7), anti-CD11c-APC (Clone HL3), and anti-CD45-PerCP-cy5.5 (Clone 30-F11), all of which were purchased from BD Biosciences.

### 4.5. Fibroblast Isolation and Culture

Kidney fibroblasts were prepared from mice as described previously [22]. In brief, the cortex of kidneys were collected separately and then minced in Petri dishes (Nunc, Roskilde, Denmark). Renal fibroblasts were cultured in Dulbecco′s modified Eagle′s medium (DMEM) and 20% fetal calf serum (FCS) (Gibco, Grand Island, NY, USA) supplemented with l-valine (46 mg/mL) (Sigma Chemical Company) and 1% penicillin-streptomycin; the cells were subsequently incubated at 37 °C under 95% air/5% CO_2_. Growth media (DMEM and 20% FCS) were replaced every other day after cultured cells could be seen in dishes (about 7 to 8 days). The cells reached confluence in Petri dishes 15–20 days after setup. By this time, subcultures were performed by separating cells into new sterile flasks. Renal fibroblasts between passages 3 and 5 were used for experiments.

### 4.6. Co-Culture Experiments

For CD8^+^ T cell-fibroblasts co-culture, 5 × 10^4^ fibroblasts from WT mice were co-cultured for 24 h with 5 × 10^3^ CD8^+^ CD11c^−^ or CD8^+^ CD11c^+^ T cells from UUO mice at day 5 after cell sorting. After a 24-h culture, the cells were collected, incubated with anti-CD8 and anti-CD3 antibody for gating out CD8^+^ T cells, and added with PI for fibroblast mortality analysis through flow cytometry.

### 4.7. Statistical Analysis

Data are expressed as means ± sem. Significance testing was performed using one-way analysis of variance followed by pair-wise comparisons using the *Student–Newman–Keuls* test. Statistical significance was set at *p* < 0.05 and *p* < 0.01. At least three replicates were performed for each experimental condition.

## 5. Conclusions

Our results suggest that different arms of the immune system will be activated to provide protection against renal injury with subsequent fibrosis at different stages. Our ureteric obstruction model showed a new role of CD8^+^ T lymphocytes, wherein they contribute to the reduction of renal fibrosis partly by inducing apoptosis of activated fibroblast.

## Figures and Tables

**Table 1:** Primer information.

mRNA	Forward	Reverse
*PRF-1*	5′-AAAAACTCCCTAATGAGAGACGC-3′	5′-ACACGCCAGTCGTTATTGATATT-3′
*GZMA*	5′-TGCTGCCCACTGTAACGTG-3′	5′-GGTAGGTGAAGGATAGCCACAT-3′
*GZMB*	5′-CCACTCTCGACCCTACATGG-3′	5′-GGCCCCCAAAGTGACATTTATT-3′
*FasL*	5′-TCCGTGAGTTCACCAACCAAA-3′	5′-GGGGGTTCCCTGTTAAATGGG-3′
*CCL2*	5′-GTCTGTGCTGACCCCAAGAAG-3′	5′-TGGTTCCGATCCAGGTTTTTA-3′
*CCL3*	5′-TTCTCTGTACCATGACACTCTGC-3′	5′-CGTGGAATCTTCCGGCTGTAG-3′
*CCL4*	5′-TTCCTGCTGTTTCTCTTACACCT-3′	5′-CTGTCTGCCTCTTTTGGTCAG-3′
*CCL5*	5′-AGATCTCTGCAGCTGCCCTCA-5′	5′-GGAGCACTTGCTGCTGGTGTAG-3′
*GAPDH*	5′-TGCCCCCATGTTTGTGATG-3′	5′-TGTGGTCATGAGCCCTTCC-3′
